# Feature extraction and selection for objective gait analysis and fall risk assessment by accelerometry

**DOI:** 10.1186/1475-925X-10-1

**Published:** 2011-01-09

**Authors:** Benoit Caby, Suzanne Kieffer, Marie de Saint Hubert, Gerald Cremer, Benoit Macq

**Affiliations:** 1 Telecommunications and teledetection lab, Université Catholique de Louvain, Place du levant,Louvain-la-Neuve, Belgium; 2 Geriatric department, Mont-Godinne University Clinics, Mont-Godinne, Belgium

## Abstract

**Background:**

Falls in the elderly is nowadays a major concern because of their consequences on elderly general health and moral states. Moreover, the aging of the population and the increasing life expectancy make the prediction of falls more and more important. The analysis presented in this article makes a first step in this direction providing a way to analyze gait and classify hospitalized elderly fallers and non-faller. This tool, based on an accelerometer network and signal processing, gives objective informations about the gait and does not need any special gait laboratory as optical analysis do. The tool is also simple to use by a non expert and can therefore be widely used on a large set of patients.

**Method:**

A population of 20 hospitalized elderlies was asked to execute several classical clinical tests evaluating their risk of falling. They were also asked if they experienced any fall in the last 12 months. The accelerations of the limbs were recorded during the clinical tests with an accelerometer network distributed on the body. A total of 67 features were extracted from the accelerometric signal recorded during a simple 25 m walking test at comfort speed. A feature selection algorithm was used to select those able to classify subjects at risk and not at risk for several classification algorithms types.

**Results:**

The results showed that several classification algorithms were able to discriminate people from the two groups of interest: fallers and non-fallers hospitalized elderlies. The classification performances of the used algorithms were compared. Moreover a subset of the 67 features was considered to be significantly different between the two groups using a t-test.

**Conclusions:**

This study gives a method to classify a population of hospitalized elderlies in two groups: at risk of falling or not at risk based on accelerometric data. This is a first step to design a risk of falling assessment system that could be used to provide the right treatment as soon as possible before the fall and its consequences. This tool could also be used to evaluate the risk several times during the revalidation procedure.

## Background

Falls in the elderly is nowadays a major concern because of their consequences on elderly general state and the global aging of the population. They are even the leading cause of injury-related visits to the emergency services in the United States and are the primary etiology of accidental death for people aged over 65 [1]. Older people with a fall experience develop usually fear of falling [2], reduction of daily activities [2,3], social isolation and morbidity [4]. Risk factors are multiple and various: e.g., visual impairments [5], reduced limbs mobility, proprioception impairment, cognitive impairments [4] or medication [6]. Moreover there are also extrinsic risks factors related to the living environment; e.g. carpets, weak lightning, cords and wires on the floor [1],... The risk of falling is generally assessed by clinical walking and standing tests such as the Tinetti test [7] or Timed up and go test [8] in addition with a global general health diagnostic. Most of these tests are clinical scores assessed by a physiotherapist or a physician and are therefore subject to human subjectivity. Moreover there exist several versions of the tests that make comparisons difficult [9]. Therefore it is important to develop an objective, simple and reproducible test to assess the risk of falling. Such a test can be used to diagnose the risk of falling and to monitor walking performances during and after rehabilitation. This paper focuses on the risk of falling related to gait patterns.

This paper proposes an objective risk of falling assessment based on accelerometric data collected when walking on a 25 m distance at comfort speed. This simple test can be made at home as well as in hospital or by the house doctor, thanks to a portable accelerometer network. Accelerometers are already used in a multitude of health related projects such as activity assessment [10], freezing of gait in Parkinson's disease [11], fall detection [12], elderly gait study [13], elderly fall prevention [14] and risk of falling assessment [15,16]. The novelty of our approach relies on the following two points:

• Use of a whole accelerometer network that acquires 3 D data from all the limbs;

• Computation of new features in this field of application;

## Method

### The accelerometer network

This section will be dedicated to the accelerometer network and the way the accelerometers are set on the body for gait analysis.

#### Hardware description

The network is composed of 10 sensors and one data logger. Each sensor is based on a Freescale MMA7261Q 3-axis accelerometer [17] and a microcontroller with 10 bits precision ADC. The sensors are connected to the data logger via 6 channels with a maximum of 8 serial-connected sensors per channel. The accelerometers have selectable sensitivity and range between ± 2.5 *g*, 3.3 *g*, 6.7 *g *and 10 *g *so that the range that best fits the kind of data to be analyzed can be selected. In this study, the chosen sensitivity is 10 *g *for all sensors to avoid saturation. The data logger communicates with the sensors via I2C protocol [18] and collects the 3 D accelerations on each sensor at 50 *Hz*. The accelerometers synchronously take measurements thanks to a dedicated broadcast line that gives the start measurement signal each 20 *ms*. Then, the data logger sequentially collects data from each sensor. Data are then stored on a 256 MB flash memory that allows recording up to 45 minutes. They are then transfered to a PC via USB port for offline processing and analysis.

The sensors are 25 × 20 × 7 *mm *big and the data logger 115 × 70 × 25 *mm*.

#### Positioning on the body

**Figure 1:**
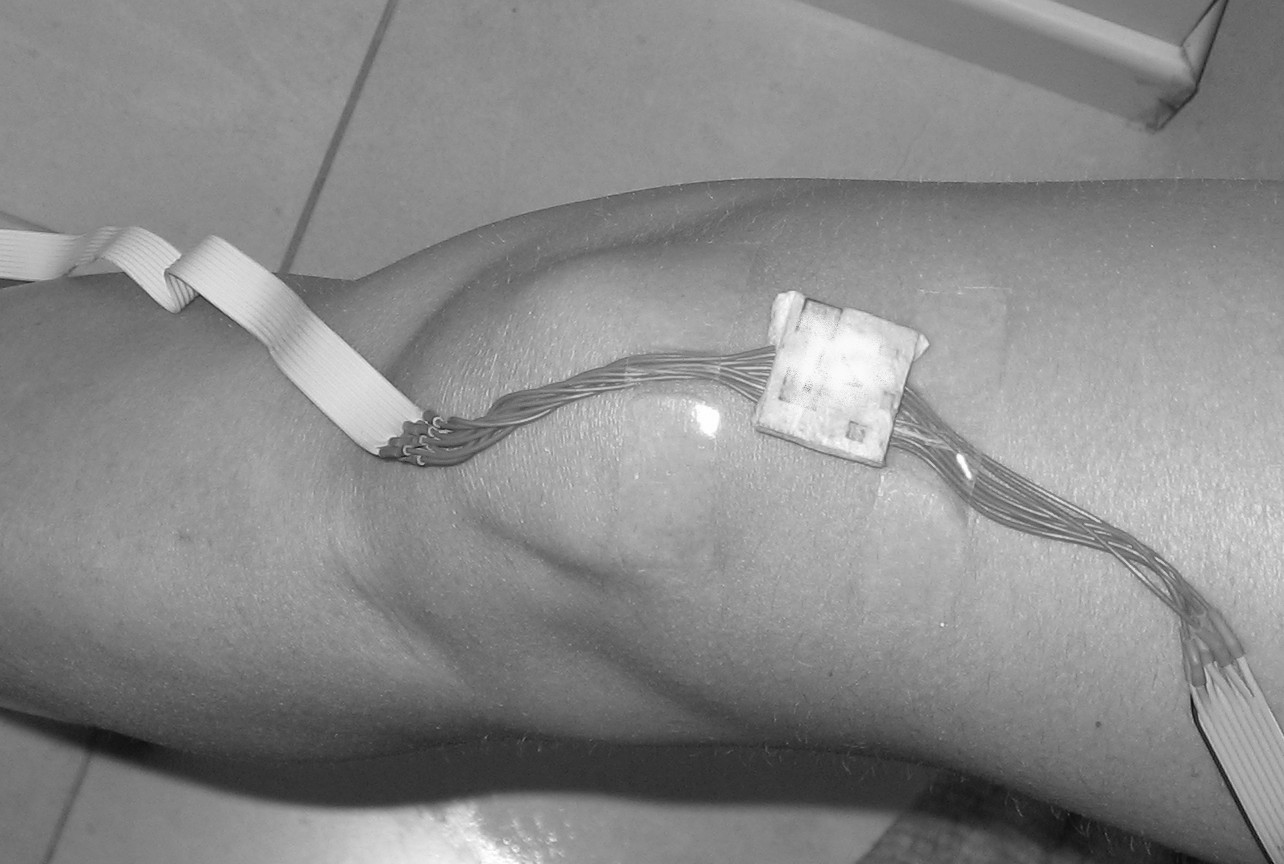
**Knee sensor**. Knee sensor position.

**Figure 2:**
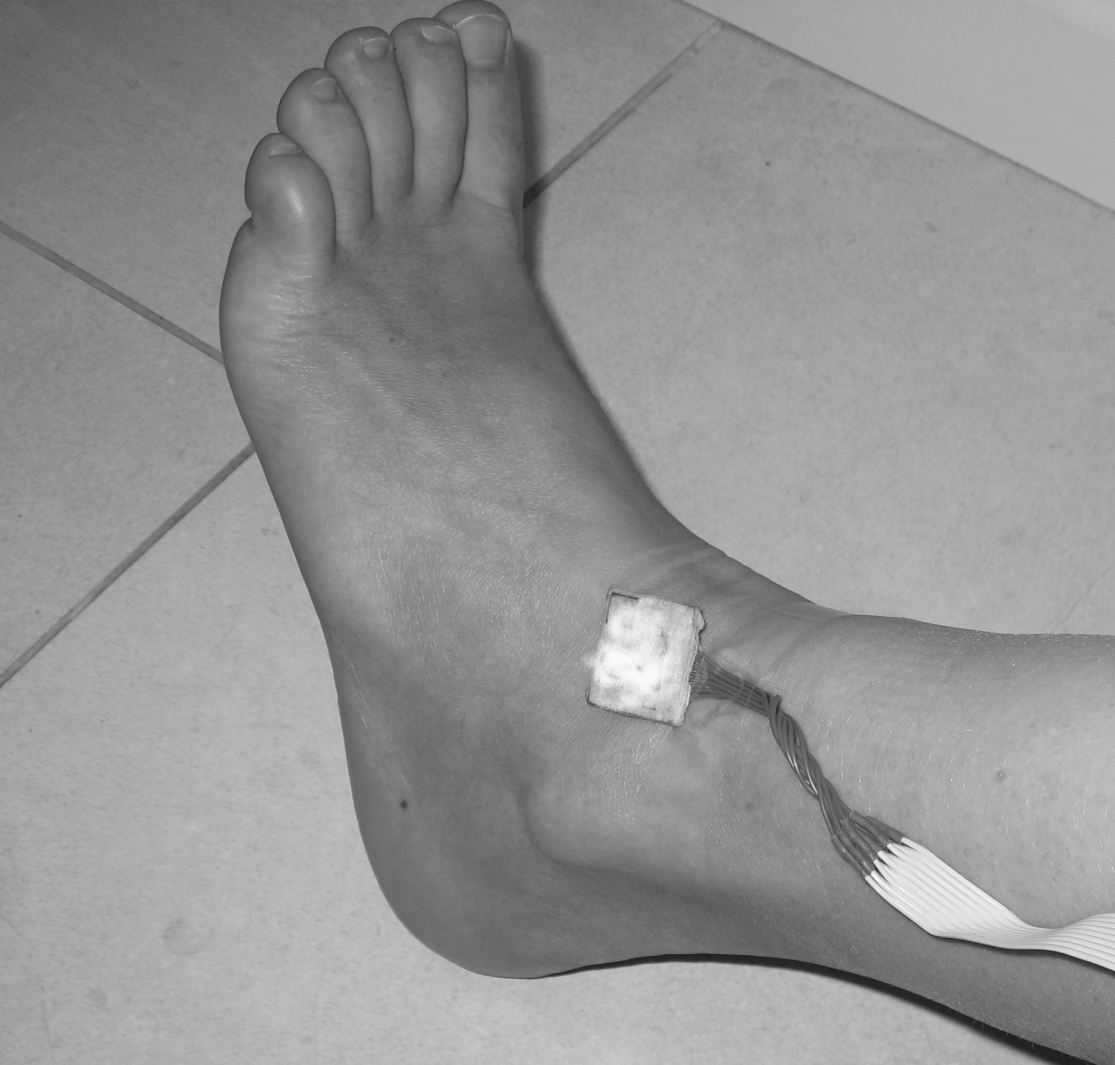
**Ankle sensor**. Ankle sensor position.

**Figure 3:**
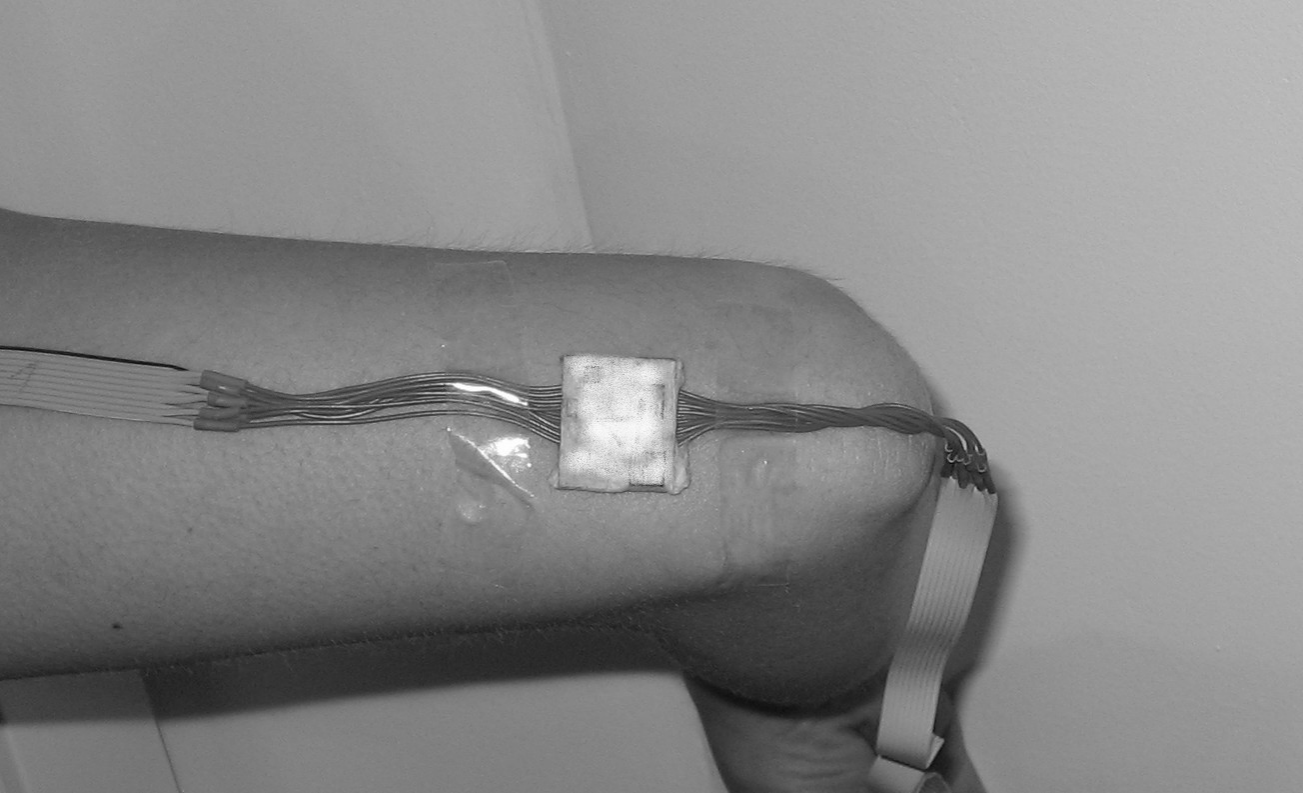
**Elbow sensor**. Elbow sensor position.

**Figure 4:**
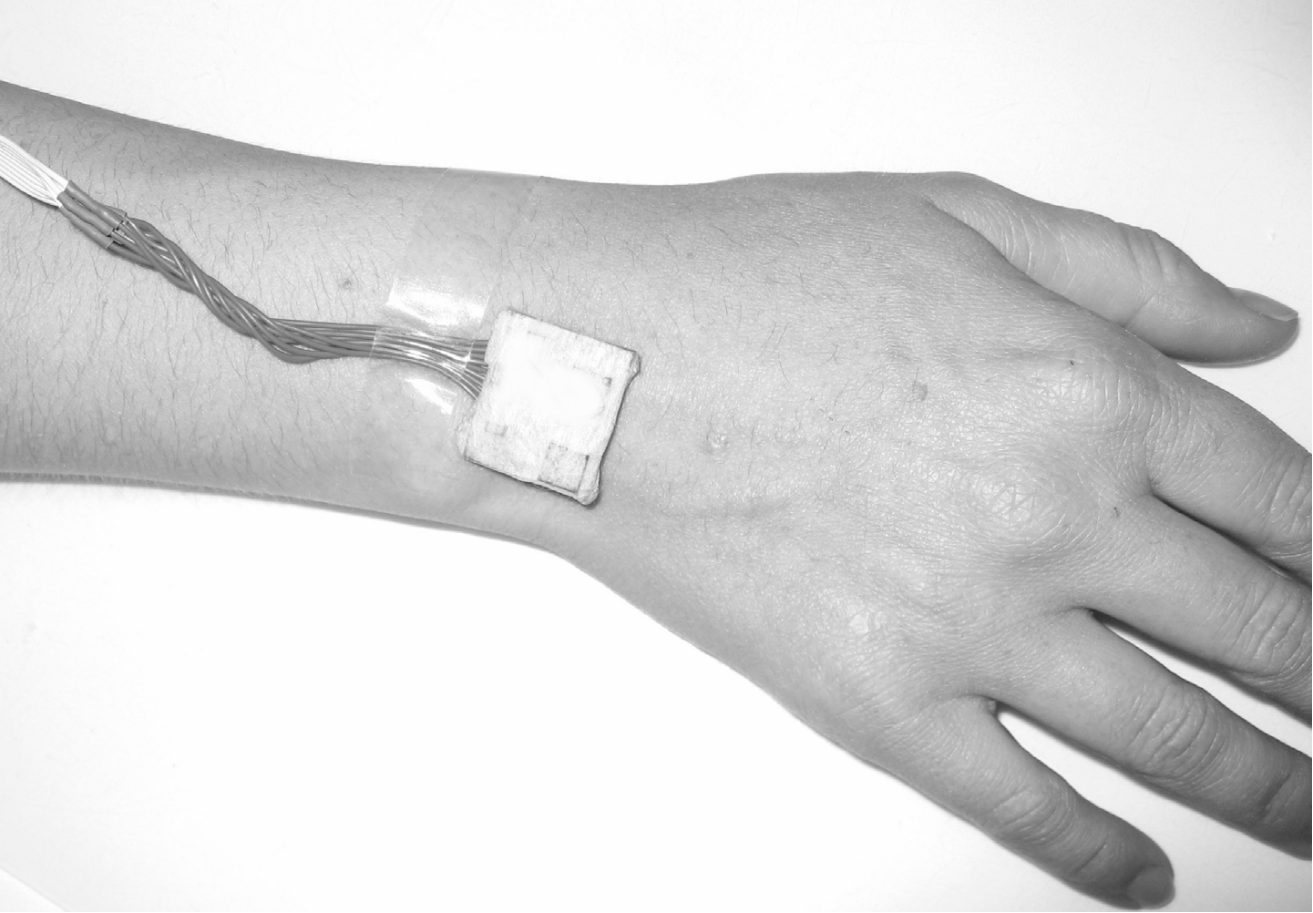
**Wrist sensor**. Wrist sensor position.

**Figure 5:**
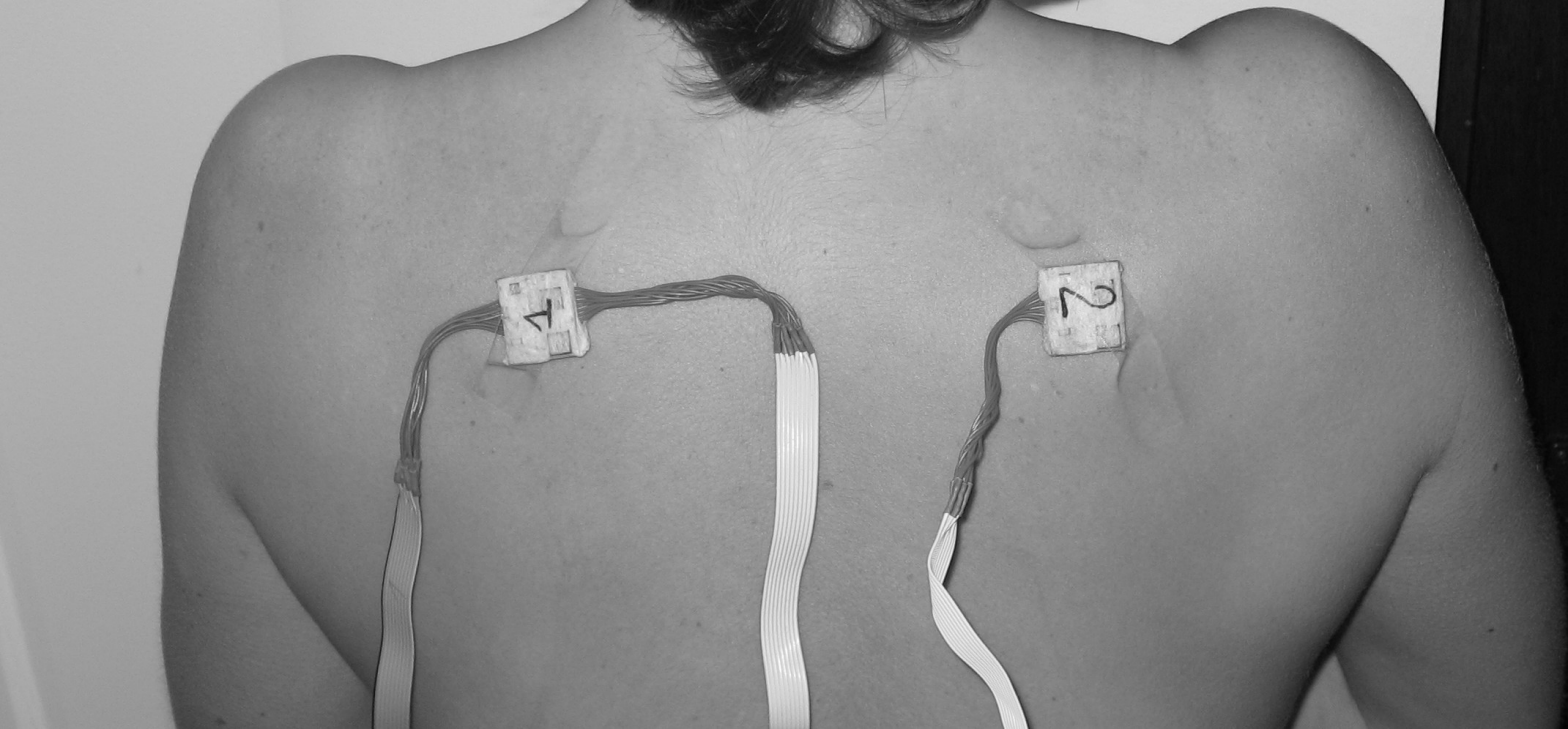
**Shoulder blades sensors**. Shoulder blades sensor position.

Sensors are set on each limb as follows:

1. left knee as showed at Figure 1

2. left ankle as showed at Figure 2

3. right knee as showed at Figure 1

4. right ankle as showed at Figure 2

5. left elbow as showed at Figure 3

6. left wrist as showed at Figure 4

7. right elbow as showed at Figure 3

8. right wrist as showed at Figure 4

9. left shoulder blade as showed at Figure 5

10. right shoulder blade as showed at Figure 5

Written consent for publication of these pictures were obtained. Sensors placed on the same limb (for example, the right knee and the right ankle) are serial-connected on the same channel to minimize wire load. They are fixed with elastic velcro straps except on the shoulder blades, where they are placed with medical tape. The data logger is fixed on a belt worn at the waist.

### Clinical study

This section will first describe the recruitment procedure and the inclusion and exclusion criteria. Then it will present some usual gait and balance assessment clinical tests. It will finally describe the clinical protocol of the study which includes the experimental procedure and the settings.

#### Recruitment

A population of 20 elderly people (14 women and 6 men) was recruited, between October 2008 and July 2009, at the geriatric department of the UCL University Clinics of Mont-Godinne (Belgium). All the subjects had to be aged over 70 years (mean: 80.85, std dev: 5.18) and had to be in stable medical condition. In addition, they all had to be able to walk on 25 m without any assistance and must have the mental abilities to follow the instructions given by the therapist. 75% of the participants were considered at risk of falling (average Tinetti score: 22.07, *σ *= 4.8, average MMT: 17.47, *σ *= 2.2) and 25% not at risk (average Tinetti score: 22.4, *σ *= 4.4, average MMT: 18.2, *σ *= 1.8). These not equal sized groups (male-female and fallers-non-fallers) reflects the geriatric departement population respecting the inclusion criteria abovementioned. This study was approved by the Ethical Committee of the hospital (University Hospital of Mont-Godinne). Each participant provided an oral informed consent.

#### Clinical tests

A complete gait and balance report was written by the physiotherapist for each patient. This report included information about previous falls, several usual clinical tests, described in this section, and recommendations for management and treatment. The risk of falling is evaluated from the conclusions of this report.

• 25 *m *walking: is a simple test that consists on walking on a 25 *m *distance at comfort speed without speaking. The subject begins standing still, starts to walk and stops at the end of the 25 m. The time needed to walk is measured by the experimenter.

• Mini Motor Test (MMT): is a 20-item score which assesses the abilities both in bed and in the standing position, and quality of both the sitting position and the gait [19]. This test also evaluates the psychomotor state and the fear of falling.

• Tinetti Test: or Performance-oriented Mobility Assessment (POMA) [7] has been claimed to be the golden standard in assessing mobility dysfunctions in the elderly and an important fall risk assessment measure [8,9]. This test assesses gait and balance in several positions. Its most frequent version is a 20-item test scored between 0 and 28: 10 items dedicated to the gait assessment (score between 0 and 12) and 10 items for balance evaluation (score between 0 and 16).

• Timed up-and-go test [8,20]: is a test measuring the necessary time to stand up from an armchair, walk 3 *m*, come back and sit down on the armchair. The time needed to execute this test assesses the locomotive dependence of the subject.

• Physical Performance Scale (PPS) [21]: evaluates the lower extremity functions by several gait, balance and stand up tasks.

• Fukuda test: consists in stepping in place with outstretched arms and blinded eyes [22]. It is used to detect vestibular impairments.

• One leg balance: the subject have to stay, unassisted, on one leg during 5 seconds. According to [23], this test assess the balance and the risk of injurious falls.

All these tests are different manners to assess the balance and the gait of the subjects.

#### Clinical Protocol

The procedure defined in this section is the clinical protocol used for this study. This protocol was approved by the ethical committee of the UCL University Clinics of Mont-Godinne before the beginning of the measurements.

Each subject was first informed about the study, and was asked to participate. After oral consent, the participant answered simple questions related to previous falls and living environment:

• Did you fall in the year? How many times?

• Do you live at home or in a rest-home?

• Do you live alone?

Height of the subject was measured. The first tests were made by the physiotherapist, in a dedicated room, one subject at a time, in the following order:

1. MMT

2. Tinetti test

3. Fukuda test

Then the accelerometer network is placed on the subject's body. This step takes around 10 minutes and allows the subject to rest during this time. All the following tests are recorded by the data logger. The recording is stopped between each test in order to write start markers in the recorded signal.

The first record is the 25 m walking test. Therefore, the subject is taken to the gerontology hall where start and stop lines are marked on the floor. The therapist has to make sure that the way is free of obstacle, has to start recording, and then has to give the start signal to the subject and has to begin to chronometer. After the stop line, the therapist stops the chrono timer and the recording. Then the therapist goes back to the dedicated room with the subject to execute the remaining test after a few minutes rest for the subject.

The second test is a recorded timed up and go test.

The third test is the PPS with a restart of the data logger between each item of the test. And finally, the last test is the one leg balance made without restart between the two legs.

After all these tests, the subject goes back to her/his room and the therapist writes the gait and balance report. The recording is transferred to a PC and saved in a text file which name is made of the initial letters of the hospital and a number increased by one for each new subject.

### Data processing

Because 3 D data were collected from 10 sensors at 50 *Hz*, it was impossible to use raw data to classify fallers and non-fallers. Therefore several features were extracted from the raw signal and were given as input to the classifier. This section will describe the computed features and present a way to select the most discriminative ones. Thereafter, several classification algorithms will be implemented with the aim to classify fallers and non-fallers. The classification performances of each algorithms will be compared.

#### Feature extraction

A total of 67 features were extracted from the 25 m walking test of each subject: The *Time to Walk *(TtW) computed between the beginning and the end of the legs motion; The *Number of Steps *(NbStep), *Step Frequency *(StepFreq) and *Variance of Step period *(VarStep) computed from legs signals; The *Median Frequency *(MF) computed for each sensor such that (1) is verified [24]. It represents the frequency under which the half of the PSD power is.

(1) $$\sum_{0}^{MF}PSD\left(f\right)=\frac{1}{2}\sum PSD\left(f\right)$$

The *Spectral Entropy *(SpecEnt) was computed as in (2) for all 10 sensors [24].

(2) $$SpecEnt=\frac{-1}{log\left(M\right)}\sum_{f=0}^{50\;H\;z}PSD_{n}\left(f\right)\;\;log\left[PSD_{n}\left(f\right)\right]$$

where *PSD_n _*is the normalization of the PSD such that

(3) $$\sum PSD_{n}\left(f\right)=1$$

The *Correlation between Arm and Leg *(CorrArmLeg) was computed as the correlation between X-axis signals from left knee and left elbow. This feature is supposed to show how the movements from leg and arm are synchronized. The *Approximate Entropy *(ApEn), a measure of the complexity of the signal, was computed for each sensor as in [25]. The *third Momentum *(M3) that measures the asymmetry of the distribution [26] and the *Peakedness *(Ma) [26] were computed for each sensor as in (4) and (5).

(4) $${\hat{m}}_{3}=1/\left(N-1\right)\sum_{t=1}^{N}x{\left(t\right)}^{3}$$

(5) $${\hat{m}}_{a}=1/\left(N-1\right)\sum_{t=1}^{N}\left|x\left(t\right)\right|$$

where *x *(*t*) represents the X-axis acceleration of the considered sensor.

**Figure 6:**
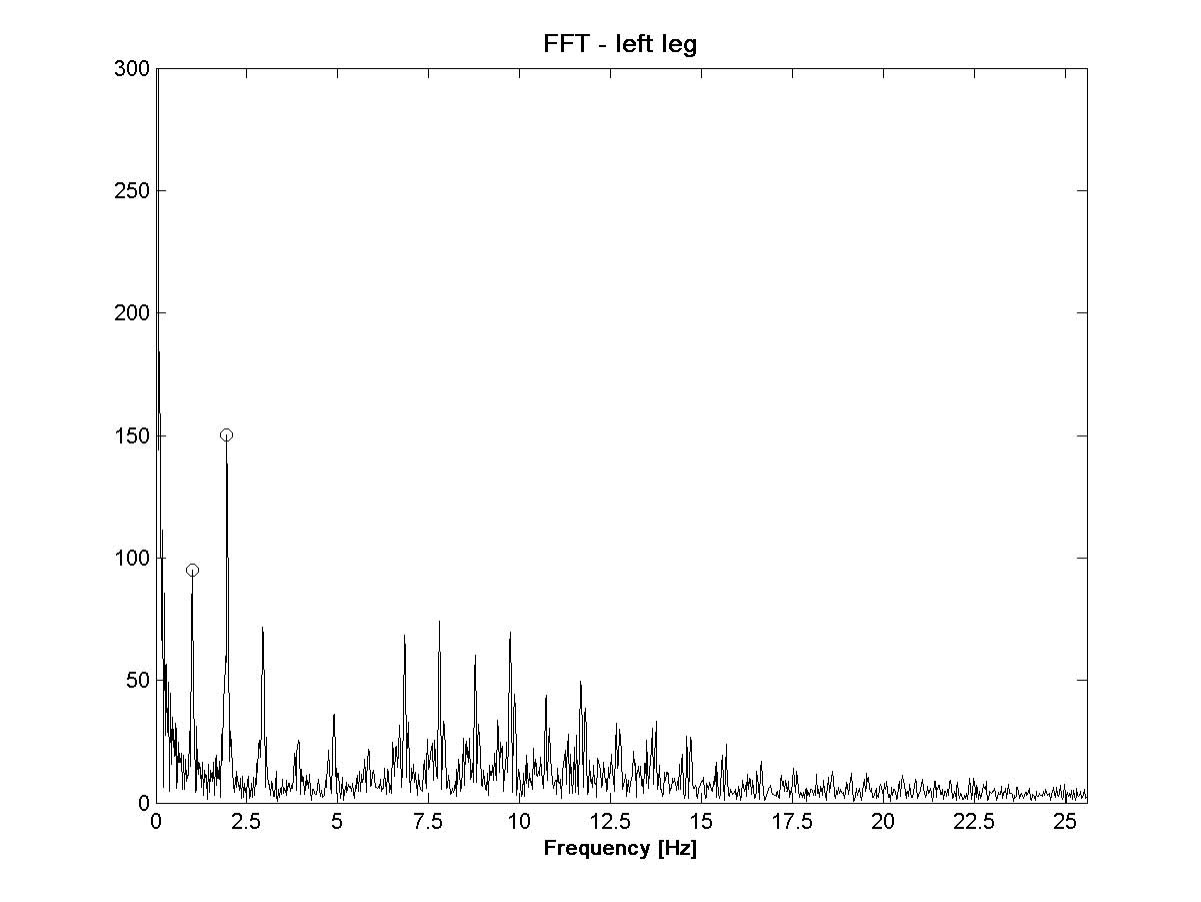
**FreqPeakX1 and FreqPeakX2**. Features 47 and 48 are the frequencies of the first and second frequency peaks highlighted by black circles. 2048 points FFT of the left leg acceleration. The FreqPeakX and FreqPeakY features are the X and Y coordinates of highlighted points.

Figure 6 shows the 2048 points FFT of the left knee signal of an aged person. This figure shows some peaks highlighted with data tips. The *coordinates of the two first peaks *were taken in the feature set as (FreqPeakX) and (FreqPeakY). The height of the patient can have an influence on some parameters like StepFreq or NbStep, therefore the *Size *had to be one of the features. For some features, it makes sense to normalize them dividing or multiplying them by the height. *Normalized Time *(Norm. Time), *Normalized NbStep *(Norm. NbStep) and *Normalized StepFreq *(Norm. StepFreq) were obtained this way. In order to detect arm motion asymmetries, the *Arm Correlation *(ArmCorr) was computed as the correlation of the X-axis of sensors fixed on the elbows; the X-axis is aligned with the arm. Another way to analyze mobility reduction of the arms is to compute the *Left Arm Dynamic *(ArmDynL) and *Right Arm Dynamic *(ArmDynR). These dynamics were computed from the norm of the accelerations of left and right elbows. The last features are approximations of the *Normalized Jerk *(NJ) for each sensor which, according to [27], assess the average movement disfluency. The NJ is defined by: (6).

(6) $$NJ=\sqrt{\frac{T^{5}}{2D^{2}}\int_{t_{0}}^{t_{end}}j^{2}\left(t\right)dt}$$

where *T *is the total movement time, *D *the total distance and *j*(*t*) is the jerk computed as the first derivative of the acceleration. Because the acceleration signals obtained by the accelerometers are composed by the dynamic acceleration and the gravity, the less the sensor is rotating, the more the NJ approximation will be accurate.

#### Significance analysis

First, the risk evaluation, made by the expert, and based on the falling experience and the scores obtained at the clinical tests above mentioned, was used as risk of falling estimation golden standard. More precisely, people who experienced a fall in the last 12 months where considered at risk. Indeed, previous falls were reported by [4] as one of the most important risk factor for future falls. Two exceptions to this rule were made: Patient 13 did not report any previous fall but his anti-falling reflexes were altered and he presented static wobble. Moreover, this patient had several prostheses and heavy cognitive impairments which let us think about forgotten previous falls. The second exception is patient 20 which reported falls due to dizzy turn. This patient scores very well for the clinical tests (Tinetti: 28, TMM: 20/20 and TUG < 20 s). These high scores encouraged us to set him in the not at risk group as this article only considers gait related risk of falling. The sample of patients is composed of 15 persons at risk aged of 80.07 (std dev: 5.34) and 5 persons not at risk of falling aged of 83.2 (std dev: 4.32).

**Figure 7:**
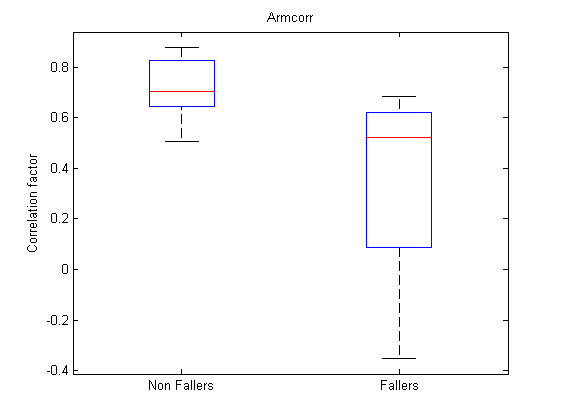
**Boxplot feature 55**. Boxplot for feature 55 (ArmCorr).

**Figure 8:**
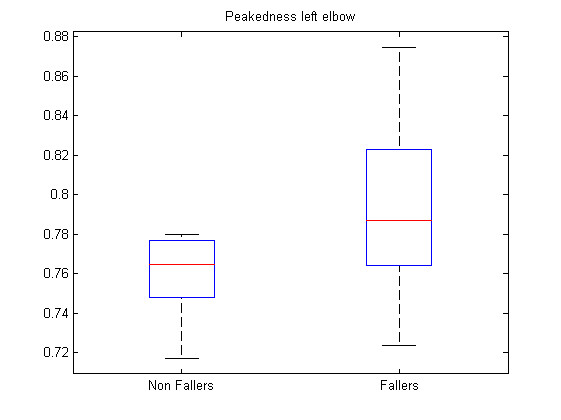
**Boxplot feature 41**. Boxplot for feature 41 (Ma left elbow).

The first analysis made is the comparison of each feature between the two groups via a t-test. Because the variances in both groups does not seem to be equal considering the sample, the problem of hypothesis testing becomes a Behrens-Fisher problem [28] which does not consider equal variances for the two groups. Using this test directly on the features gives the lowest p-values for features 55 (*p *= 0.002) and 41 (*p *= 0.037) of Table 1. Figure 7 and Figure 8 show the boxplots of features 55 and 41 respectively. In addition, following the intuition that some features should have an ideal value and that a large deviation from this value could reflect abnormal walking patterns; intuition that is confirmed by the larger variances for almost every features for the fallers group; t-tests were made on the distance to the mean of each feature. Equation (7) shows the transformation applied on each feature tested.

**Table 1:** Features numbers, labels and short descriptions

**Feat. no**.	Name	Description
1	TtW	Time to Walk 25 m at comfort speed
2	NbStep	Number of step to walk 25 m
3	StepFreq	Frequency of the Steps
4	VarStep	variation of the step period
5-14	MF	Median Frequency (sensors 1-10)
15-24	SpecEnt	Spectral entropy (sensors 1-10)
25	CorrArmLeg	Correlation between Arm and Leg
26	ApEn	Approximate entropy for sensor 1
27-36	M3	Third momentum (sensors 1-10)
37-46	Ma	Peakedness (sensors 1-10)
47	FreqPeakX1	Abscise of the 1*^st ^*freq. peak (left leg)
48	FreqPeakX2	Abscise of the 2*^d ^*freq. peak (left leg)
49	FreqPeakY1	Ordinate of the 1*^st ^*freq. peak (left leg)
50	FreqPeakY2	Ordinate of the 2*^d ^*freq. peak (left leg)
51	Size	Size (height) of the patients in m
52	Norm. Time	TtW/Size
53	Norm. NbStep	NbStep*Size
54	Norm. StepFreq	StepFreq*Size
55	ArmCorr	Correlation between both elbows
56	ArmDynL	Dynamic of the Left Arm signal
57	ArmDynR	Dynamic of the Right Arm signal
58-67	NJ	Approx. Normalized Jerk (sensors 1-10)

(7) $$f_{i,j}=\left|f_{i,j}-mean_{i}\left(f_{i,j}\right)\right|$$

**Figure 9:**
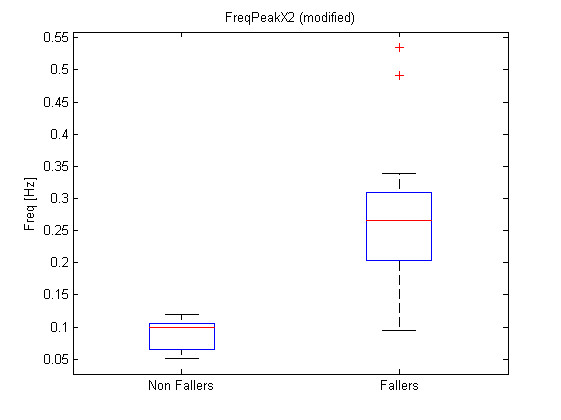
**Boxplot for modified feature 48**. Boxplot for modified feature 48 (FreqPeakX2).

**Figure 10:**
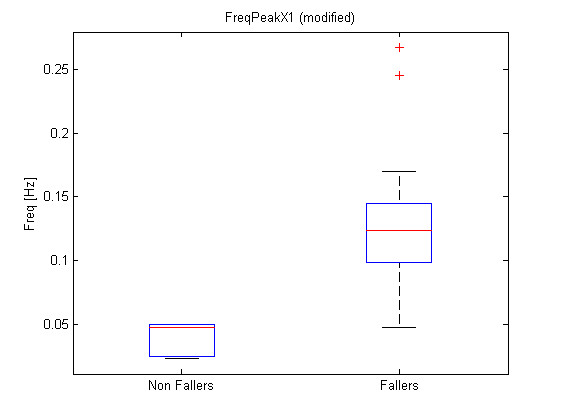
**Boxplot for modified feature 47**. Boxplot for modified feature 47 (FreqPeakX1).

**Figure 11:**
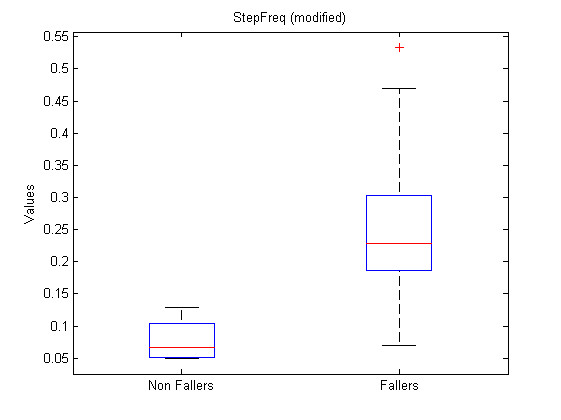
**Boxplot for modified feature 3**. Boxplot for modified feature 48 (StepFreq).

Where a *i *subscript represents the patient while a *j *subscript represents the feature number as referenced in Table 1. The list of transformed features leading to the lowest p-values and associated p-values, regrouped by sensor, is presented at Table 2. Figure 9, Figure 10 and Figure 11 show the boxplots for modified features 48, 47 and 3 respectively.

**Table 2:** Significant modified features

**Feat. no**.	Name	Sensors	p-value
1	TtW	-	0.041
3	StepFreq	-	4.35e-5
5,7,8,9,10	MF	1,3,4,5,6	[0.023-0.042]
15,17,18,19,20,22	SpecEnt	1,3,4,5,6,8	[0.021-0.045]
39,45	Ma	3,9	0.004 0.014
47	FreqPeakX1	1	0.29e-4
48	FreqPeakX2	1	0.16e-4
49	FreqPeakY1	1	0.027
52	Norm. Time	-	0.012
54	Norm. StepFreq	-	0.004
57	ArmDynR	7	0.013
60,61,66	NJ	3,4,9	[0.012 0.035]

#### Feature selection and classification

The feature selection step, in classification algorithms design, is the step where a subset of the computed features is chosen. This choice is made in order to, on the one hand, design simpler algorithms, and, on the other hand, avoid the over fitting. An over fitted algorithm is a complex algorithm that really well fits the training dataset, but provides bad results in generalization with new features vectors. The aim of this section is to present how the features were selected in order to classify elderly in two groups: the fallers (persons at risk for falling) and the non-fallers (persons not at risk); and to present algorithms able to do such a classification.

Four classification algorithms were used to select the features according to classification performances: a radial basis function network classifier (rbnc) [29], a support vector classifier (svc) [29], a k-nearest neighbors classifier (knnc) and a naive bayesian classifier (naivebc).

Basically, an rbnc is a classifier based on a neural network with radial basis functions as kernels. In the present case, a 3-neuron hidden layer was used with gaussian kernels.

An svc is a classifier that projects the feature space onto a higher dimensional space in which the dataset becomes linearly separable. It searches then an hyperplane in the created data space such that the margin between the hyperplane and both classes is maximized. Gaussian radial basis functions were used as mapping functions between the feature and the created spaces.

An knnc is a simple algorithm comparing a sample with its k-nearest neighbors and classifying it in the more represented class amongst those k-neighbors. In the present case, k was arbitrarily chosen equal to 3, and the euclidean distance is used to identify the nearest neighbors.

An naivebc is a simple probabilistic classifier based on the Bayes rule assuming independence of the features given the class. Several studies discuss over this strong independence assumption and show optimality [30] or surprisingly good performances even when this assumption is violated [31].

The performance evaluation is made by the cross-validation classification error via a leave-one out cross-validation algorithm [29]. The leave one out cross-validation procedure consists in performing the learning step with all but one sample and then testing the learned algorithm with the one remaining sample. This procedure was repeated *N *times, with *N*, the number of samples in the dataset.

The feature selection procedure used belongs to the forward wrapper selection algorithm family, which has the advantage to be particularly computationally advantageous and robust against overfitting [32]. The used feature selection algorithm begins to test performances for each feature separately, and select the ones minimizing the classification error. Then, for all these selected features, the algorithm tests the pairs made by one of these features and all the other features, and selects the pairs minimizing the error. If this error is strictly lower than the error for the single feature, the algorithm continues investigating this pair in addition with all possible non-selected features. The algorithm stops when the error at step *k *+ 1 is is not strictly smaller than the error at step *k*. This algorithm is applicable to all the considered classification algorithms.

## Results and discussion

The significance analysis made in this study is a multiple testing procedure. In this case, one can not consider significant differences for each feature leading to a p-value under *α *= 0.05. Indeed, with such a threshold, one can expect to make 1 error each 20 tests rejecting the null hypothesis. In this study, 134 tests were computed, which should statistically lead to 6.7 errors considering the 0.05 threshold. Therefore a Holm correction were applied to the obtained p-values. With such corrected p-values, the null hypothesis can be rejected for modified features 48, 47 and 3. Their corrected p-values are respectively 1.6*e *- 5 * 134 = 2.1*e *- 3, 2.9*e *- 5 * 133 = 3.9*e *- 3 and 4.35*e *- 5 * 132 = 5.7*e *- 3. One should notice that only three features are significant considering the Holm correction while 26 had a p-value < 0.05 and that statistically, only 6.7 non significant features should have a p-value < 0.05. Further attention should be taken to the other features with p-values < 0.05 to check their significance and particularly to direct feature 55 which is the first feature not respecting the Holm criteria. This feature is the ArmCorr and can reflect unnatural arm movement if low.

As for the features significance, the direct features and a modified version of the features will be proposed to the feature selection algorithm with each classification algorithm. Table 3 shows the selected features for the four classification algorithms. The first column shows common features for several feature sets, the second one shows different possibilities for the last feature, separated by a semicolon. The third column gives the classification error rate for these feature sets and the fourth and fifth columns give the sensitivity and specificity. Feature sets leading to an error bigger than a threshold depending of the cardinality of the feature set considered were omitted for the naivebc for concision purpose. These thresholds were set to 0 for cardinality 4 and 0.05 for cardinality 3; feature sets of cardinality over 4 were all omitted to avoid overfitting.

**Table 3:** Selected features

First features	Last feature	Error	Sensitivity	Specificity
**rbnc**				
47	48	0.05	1	0.8

**svc**				
	3-49; 51; 54-66	0.25	1	0

**knnc**				
45	47; 48	0.05	0.93	0.8
3		0.1	0.93	0.8

**Naivebc**				
55, 3, 52	14; 15; 35; 43	0	1	1
55, 67, 38	6; 8	0	1	1
55, 52, 66	6; 8	0	1	1
55, 52, 67	6; 8	0	1	1
55, 3	12; 51	0.05	1	0.8
55, 47	6; 8	0.05	0.93	1
55	44	0.15	1	0.4

**Figure 12:**
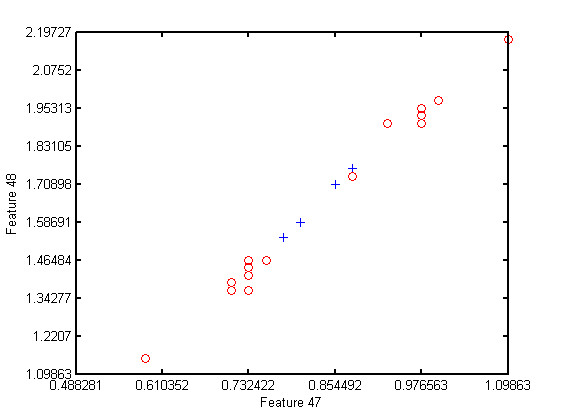
**Scatter plot for feature set 47 - 48**. Scatter plot for feature set 47 - 48, the fallers are represented by red circles while the non-fallers are represented by blue plus.

**Figure 13:**
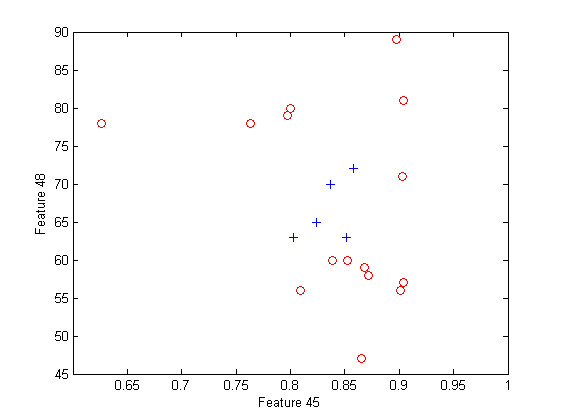
**Scatter plot for feature set 45 - 48**. Scatter plot for feature set 45 - 48, the fallers are represented by red circles while the non-fallers are represented by blue plus.

Table 3 shows that the naivebc outperforms the three other algorithms in terms of classification error, sensitivity and specificity but uses three or four features whereas the others only use one or two. This could reflect more overfitting for the naivebc. The feature 47 is selected either as unique feature, or in addition to other features for all the algorithms except for the naivebc. This feature represents the major frequency of the signal of the left leg, corresponding to the stride frequency. By definition a complete stride includes two steps, one left and one right. The features 48 and 3, which respectively represent the second frequency peak and the step frequency, are highly correlated to each other and to feature 47. One can see that the knnc performs well, even with small feature sets, but, only when one of these features is present. These characteristics were also discriminant for the modified feature of previous section. The feature 55, which measures the correlation between motion of both arms, is present in all the feature sets for the naivebc. This feature was the direct feature leading to the minimum p-value in previous section. Not surprisingly, features 6 and 8 seem to be equivalent. These features corresponds to the MF of both ankles and are therefore highly correlated (*r *= 0.998). Figure 12 and Figure 13 represent scatter plots for feature sets (47, 48) and (45, 48). Feature sets of dimension above 2 are not clearly graphically representable.

The modified features were also investigated during the variable selection step. In this case, the mean used in (7) is the mean on the training set, which is composed by all but one samples according to the leave-one-out cross validation procedure. Table 4 shows the selected features and associated errors, sensibility and specificity for the modified features. For concision purpose, feature sets containing more than four features were omitted for all algorithms. In addition, the feature sets leading to an error above a threshold depending on the cardinality of the set were also omitted for the naivebc algorithm. The thresholds were set to 0.05 and 0.1 for cardinalities 4 and 3 respectively.

**Table 4:** Selected modified features

First features	Last feature	Error	Sensitivity	Specificity
**rbnc**				
29	27	0.1	1	0.6
25		0.2	1	0.2

**svc**				
	1-48;51;53-67	0.25	1	0

**knnc**				
11		0.2	0.93	0.4
29		0.2	1	0.2
55		0.2	1	0.2

**naivebc**				
21,33,43	2;28	0.05	1	0.8
2,27,55	57	0.05	1	0.8
3,27,32	55	0.05	0.87	0.8
21,32,57	62	0.05	1	0.8
27,55,57	25;29	0.05	1	0.8
49,55,57	62	0.05	1	0.8
32,50	10;57	0.1	1	0.6
21,33	19;20	0.1	1	0.6
32,55	19;20	0.1	1	0.6
42,57	19;20	0.1	1	0.6
21,27	57	0.1	1	0.6
21,33	39;47;48;56	0.1	1	0.6
32,55	22;47;48;61	0.1	1	0.6
42,43	57	0.1	1	0.6
14	55	0.15	1	0.4
7	33	0.2	0.93	0.4

Even if there were more significant modified features than direct features, the algorithms trained on the modified features do not show better performances than those trained on the direct features. Indeed, the rbnc does not perform as well with the modified features as with the direct ones, and presents a lower specificity. The knnc trained on the modified features gives also lower performances than with the direct features. The svc performs equivalently with the modified features, and gives bad results, considering that a classification of all patients as fallers results to an error rate of 0.25 and zero specificity. Finally, the naivebc does not perform as well for the modified features than for the direct features but is the best performing algorithm for the modified features.

## Conclusions

The developed tool computed a large set of features, summarized at Table 1, and showed significant differences between the two groups of hospitalized elderly people; i.e., the group presenting a risk of falling and the one not presenting such a risk. Features 55 (ArmCorr) was the direct feature leading to the lowest p-value. The modified features considered significant are 48 (FreqPeaxX2), 47 (FreqPeakX1)and 3 (StepFreq). The modified features represent the deviation of these features from their mean values and are computed by (7). There were a lot more modified features leading to a small p-value than direct features; but the classification algorithms did not perform better with these modified features.

The ArmCorr, representing the correlation between both arms, seems to be of great interest. Indeed, this feature appears in each feature set of the naivebc classifier for the direct features and was the most significant feature via the t-test. Moreover, only a few, studies already analyze the influence of upper limb movement and more particularly, the correlation between arms on falls in the elderly. A low correlation between arm accelerations can be related to an unnatural arm movement during the walk which can be a consequence of a more global lower limb or gait impairment.

Features related to the step frequency (features 3, 47, 48) seem also to have important discriminant power as well for the direct features as for the modified ones (considering their p-values) even if the direct features were not significant for the t-test which compares the means of the distributions.

ArmCorr and frequency related features should be further investigated to confirm the tendency observed on the sample considered in this study. Moreover a prospective study should be conducted to verify the relation between these features and risk of future falls.

As only a few features from sensors 9 and 10 seem to be of interest, and as these sensors are the most difficult to set because they are placed under the shirt with tape, future work will not include these sensor anymore. Further investigations should be done to find a minimal sensor subset leading to fair classification performances.

These results were obtained from older patients hospitalized in geriatric department, of whom 75% presented falls. Further research is needed to assess the clinical usefulness of the described features in community-dwelling seniors.

## Competing interests

To the best knowledge of the authors, no competing interests exist.

## Authors' contributions

BC performed and implemented the feature extraction and selection, took part to the experimental design and draft the text. SK took part in the data analysis and provided valuable revisions to the manuscript. MdSH and BM took part in the experimental design and revision of the manuscript procedure. GC took part in the experimental design and the data acquisition as well as in revision of the manuscript. Finally, all the authors have approved this final draft of the manuscript
